# Production, comprehension, and synthesis: a communicative perspective on language

**DOI:** 10.3389/fpsyg.2013.00233

**Published:** 2013-05-02

**Authors:** Michael Ramscar, Harald Baayen

**Affiliations:** Department of Linguistics, University of TübingenTübingen, Germany

MacDonald (2013) presents a strong case for speech production having a pivotal role in the cognition of language processing. Experimental research has been strongly biased toward the study of language comprehension, and it is an intellectual pleasure to be invited to rethink the consequences of the constraints imposed by speech production on both the form of utterances and how utterances are produced and understood.

Yet, it seems to us that production and comprehension are much more in balance. For instance, when Latin lost many of its inflectional exponents and morphed into what is now modern French, the pronouns of Latin, which were used for emphasis only, became obligatory. This, it would seem, serves the listener rather than making life easier for the speaker. In the convection cycle of language change over time, speakers time and again opt for articulatory simpler forms whenever they can. In French, this led to reduced forms (compared to Latin) of the subject and object pronouns. In modern colloquial French, these pronouns can even become prefixoids that are fusing into the verb, leading to structures such as *Jean il l'a vue Pierre.* The result is an inflectional system with subject and object marking on the verb, remarkably similar to the forms of Amerindian languages (Vendryes, 1921; Lambrecht, 1981). Simplification by the speaker is followed by diversification for the listener, which is followed by simplification by the speaker. Crucially, in the negotiation of communication, utterances only have a chance of being replicated (in the evolutionary sense) if they are both producible and understandable (cf. Steels, 1998; Steels and Wellens, 2006).

However, rather than attempting to evaluate MacDonald's program by means of individual case studies, in this commentary we take a step back, and argue for a view in which the forces of production and comprehension are not only much more balanced, but in which they are essentially the same. To understand why we think the similarities are much more important than the differences, we turn to learning theory and information theory.

As MacDonald emphasizes, *learning* is a ubiquitous aspect of experience. Although, it is often conceptualized abstractly as a process that increases knowledge (like adding entries to an encyclopedia) and that improves performance (by increasing counters in the head, whether conceptualized as Bayesian priors or by serial search in a frequency ordered encyclopedia), it is important to note that the mechanistic picture of learning that has emerged from many lines of inquiry in the cognitive and brain sciences is *discriminative*. At both low- (e.g., O'Brien and Raymond, 2012) and high- (e.g., Ramscar et al., 2013b) levels of abstraction, learning is a process that reapportions attentional/representational resources in order to maximize future predictive success (e.g., Rescorla and Wagner, 1972; Pearce and Hall, 1980; Sutton and Barto, 1998; McLaren and Mackintosh, 2000; Schultz and Dickinson, 2000; Kruschke, 2001; Danks, 2003). Prediction error is used to *discriminate* against uninformative cues and to reinforce *informative* cues. These models of learning belong to a broad class of discriminative algorithms, along with the overwhelming majority of biologically based learning models (Schultz, 2006).

An important, though little-mentioned feature of this kind of learning is that it yields an inherently lossy form of coding (Ramscar et al., 2010). If languages are learned discriminatively, the representations of relationships between form and meaning that learners acquire from experience will be subject to constant change, and these changes will involve information loss. Learned relationships between forms and meanings will be subject to constant variation, both across different language users, and within language users over time (Ramscar et al., 2013d). As MacDonald rightly observes, in these circumstances, *all* linguistic communication can be expected to involve ambiguity.

A crucial consequence of lossy coding is that linguistic forms do not simply serve as hash codes for mapping form onto meaning. The forms of language are simply not rich enough data structures to formally encode the full richness of the experiences they serve to communicate (Ramscar et al., 2010). It is therefore not at all clear what it means to say, as MacDonald does, that “linguistic utterances clearly differ from other actions in that they have both a goal (e.g., to communicate) and a meaning.” Given what we understand about learning and encoding (see Grünwald and Vitányi, 2003 for an introduction to coding theory), it is clear that utterances neither *encrypt* their meanings, nor do they map onto them in a compositional, or even determinate, way. In spite of the pervasiveness of the structural metaphor (Lakoff and Johnson, 1980) that language is like a conveyor belt transporting boxes with meanings from speaker to listener, and that it is desirable to optimally stack the boxes so that their load is uniformly distributed over the conveyor belt (Hale, 2006; Levy, 2008; Jaeger, 2010; see Ferrer-i-Cancho and Moscoso del Prado Martín, 2011; Pellegrino et al., 2011 for critiques) there is good reason to believe that meaning is not *in* the words nor *in* the sentences.

This is where Shannon (1948)'s mathematical theory of communication provides insight:

“The fundamental problem of communication is that of reproducing at one point either exactly or approximately a message selected at another point. Frequently the messages have meaning; that is they refer to or are correlated according to some system with certain physical or conceptual entities. *These semantic aspects of communication are irrelevant to the engineering problem*. The significant aspect is that the actual message is one selected from a set of possible messages. The system must be designed to operate for each possible selection, not just the one which will actually be chosen since this is unknown at the time of design.” (Our emphasis.)

In other words, whatever the experiences and goals we wish to communicate might be, a signal should not be assumed to be a compositional deconstruction of them. Instead, an encoding simply needs to enable senders and receivers to discriminate between experiences and goals *on the basis of a shared code*. For example, in a world with just two experiences (being hungry; being satiated) and no noise, a code with just two non-decompositional signals, 0 and 1, suffices.

The relationship between signals and meanings in this kind of system can be summarized as follows (MacKay, 2003):

1. A communication *system* requires a sender and a receiver to be in possession of a source code defining the scope of the possible messages that can be transmitted.
2. Communication across the system is not concerned with the *meaning* of messages. In a Shannon system the receiver *reconstructs* the source message from the received signal by *discriminating* the source message from other possible messages that might have been selected and noise introduced by the communication channel.
3. The receiver does not interpret or expand on the source message. It simply reconstructs it at the destination with no loss of signal content. In linguistic terms, necessary condition for successful communication is that a listener be able to correctly identify the form of the message sent. To the extent that a speaker and listener's codes converge, this will serve to reduce, or even eliminate, a listener's uncertainty about the experiences and goals that led a speaker to select that message, aligning the listener's predictions with the speaker's intentions.

Although, this picture is *very* different to most historical approaches to language (Frege, 1892; Russell, 1905; Wittgenstein, 1947; Miller, 1951; Chomsky, 1957, 1997; Tomasello, 2005), there are many reasons to believe that Shannon's theory provides a fruitful framework for the understanding of human communication.

First, as we noted above, learning is a process that leads to the acquisition of exactly the kind of predictive, discriminative codes that information theory specifies for artificial systems (Hentschel and Barlow, 1991; Atick, 1992). The critical difference between human and artificial communication systems is that human communicators *learn* as they go. Indeed, an alternative description of the goal of utterances is that speakers intend listeners to learn something from them. Virtually all utterances—even, “Hello!”—are intended to reduce a listener's uncertainty, whether about the world, or the thoughts, feelings etc., of a speaker; learning is largely defined in terms of this kind of uncertainty reduction (Rescorla, 1988; Hentschel and Barlow, 1991; Ramscar et al., 2013b).

Second, since learning is a discriminative process, acquiring a language amounts to learning how forms discriminate between the rich experiences and goals that speakers and listeners share (see Baayen et al., 2011, for a proof of concept). From this perspective, MacDonald's suggestion that prediction serves to “guide comprehension,”—somehow helping rich semantic understandings to be mysteriously extracted from a few sparse signals (Ramscar, 2010)—is unnecessarily vague and complicated when compared to a more straightforward view of comprehension as the reduction of listeners' uncertainty about speakers' intentions as messages unfold (Ramscar et al., 2010; see also Pickering and Garrod, 2007; McMurray and Jongman, 2011).

Third, not only does learning appear to extract a particular kind of predictive code (Schultz and Dickinson, 2000), but the distributional structures of languages correspond closely to *optimal predictive codes* (Hentschel and Barlow, 1991). In Shannon entropy terms, the least efficient possible code has a uniform distribution (i.e., one in which all alternatives are equiprobable at any given choice point) and the most efficient code is one in which items are distributed in the most non-uniform way possible (i.e., a power law distribution). The distributions of languages approximate the latter at every level so far examined (Zipf, 1949; Genzel and Charniak, 2002, 2003; Aylett and Turk, 2004, 2006; Manin, 2006; Futrell and Ramscar, 2011; Ramscar and Futrell, 2011; Piantadosi et al., 2011).

Finally, it is clear that the nature of learning changes across childhood (Ramscar and Gitcho, 2007; Thompson-Schill et al., 2009; Ramscar et al., 2013c). Very young children are deficient in many prefrontal functions that, as MacDonald emphasizes, are important to speech planning. This is a curious adaptation, but it offers at least one benefit: if “simple” discriminative learners are exposed to a highly structured environmental stimulus—a language and its experiential correlates—and are restricted to sampling it in the same, non-deliberative way, they will learn very similar *systems* of mappings (Ramscar et al., 2013a; see also Shannon, 1956).

In other words, learning, and its developmental trajectory across childhood, are particularly well-adapted for the acquisition of *common* predictive codes (in the Shannon sense), and linguistic distributions appear to have evolved—socially—to optimize these codes for communication (in the Shannon sense). It is within this information-theoretic rethinking of language that the question of the relative importance of comprehension and production in shaping language comes to stand in a different light.

We immediately acknowledge that linguistic distributions must be optimized for speech production (see also Zipf, 1949). However, we contend that this optimization is totally constrained by what the listener can tolerate. For instance, in spoken Dutch, the word *eigenlijk* (actually) can reduce to *egk*. However, the speaker cannot opt for articulatory laziness in total disregard of the listener. Native speakers of Dutch do not understand *egk* when spoken in isolation (Ernestus et al., 2002; Kemps et al., 2004), and successful comprehension critically depends on its use in appropriate contexts. In other words, *egk* is a functional element of the speech signal by the grace of being part of a code that speakers and listeners share. Thanks to this shared code, what is easy for the speaker to produce is easy for the listener to understand. Likewise, what is more difficult for the speaker to encode, at whatever level of linguistic structure, is more difficult for the listener to decode. These considerations lead to the prediction that for each of the interesting examples discussed by MacDonald where we currently see optimization for production at work, there is a corresponding benefit for comprehension. If, as we suspect, Shannon's view of communication is correct, these benefits *must* be there, even if it is difficult to discern them at present, given our still very limited understanding of the experiences, and their neuro-cognitive instantiations, that we share when communicating with language.
